# Hematoxylin and eosin staining of intact tissues via delipidation and ultrasound

**DOI:** 10.1038/s41598-018-30755-5

**Published:** 2018-08-16

**Authors:** Yawu Li, Ning Li, Xiang Yu, Kai Huang, Ting Zheng, Xiaofeng Cheng, Shaoqun Zeng, Xiuli Liu

**Affiliations:** 1 Britton Chance Center for Biomedical Photonics, Wuhan National Laboratory for Optoelectronics-Huazhong University of Science and Technology, Wuhan, Hubei 430074 China; 2 MoE Key Laboratory for Biomedical Photonics, Collaborative Innovation Center for Biomedical Engineering, School of Engineering Sciences, Huazhong University of Science and Technology, Wuhan, Hubei 430074 China; 3 Convergence Technology Co., Ltd., Wuhan, 430074 China; 4 Wuhan OE-Bio Co., Ltd., Wuhan, 430074 China

**Keywords:** Biochemistry, Biological techniques

## Abstract

Acquiring information on the precise distribution of a tumor is essential to evaluate intratumoral heterogeneity. Conventional hematoxylin and eosin staining, which has been used by pathologists for more than 100 years, is the gold standard of tumor diagnosis. However, it is difficult to stain entire tumor tissues with hematoxylin and eosin and then acquire the three-dimensional distribution of cells in solid tumors due to difficulties in the staining and rinsing. In this paper, we propose a modified hematoxylin and eosin staining method, in which delipidation and ultrasound waves were applied to enhance tissue permeability and accelerate dye diffusion. This improved hematoxylin and eosin staining method is termed iHE (intact tissue hematoxylin and eosin staining). We applied the iHE method to stain intact organs of mice, which were then sectioned and imaged sequentially. The results showed that the whole tissue was stained homogeneously. Combined with micro-optical sectioning tomography (MOST), the iHE method can be used for 3D volume imaging and to evaluate the intratumoral heterogeneity of the entire tumor tissue spatially. Therefore, this method may help to accurately diagnose the invasion stage of tumors and guide clinical treatments.

## Introduction

For visualization of lesions in tissues, many techniques have been developed and can be divided into two categories. One category includes histochemical techniques^1^, such as immunohistochemistry, immunofluorescence, *in situ* hybridization and hematoxylin and eosin staining (H&E staining). For biopsy samples, these techniques can reveal specific chemical components of cells and provide subcellular structural information of lesions. However, it can be labor intensive to present the overall distribution of cells in lesions. The other category is medical imaging^2^, such as ultrasound imaging, positron emission tomography, magnetic resonance imaging and X-ray imaging^3,4^. These techniques can examine dynamic changes at a large scale, but it is hard to achieve single-cell resolution. Thus, it is difficult to distinguish individual cells in lesions at a large scale.

In the last decades, techniques for volume imaging have been developed^5–17^, achieving single-cell resolution in a labeled tissue^18^. Tissue clearing-based volume fluorescence imaging represents one such technique^19^, in which cellular nuclei are stained and each cell can be located^4,20,21^. These techniques can be used to study cancer in mouse models or to evaluate human biopsy samples, but these images are not natural H&E images, and only the latter is the gold standard to diagnose tumors. If an intact tumor tissue can be stained with H&E, and volume bright-field imaging can be applied to the stained sample, volume imaging can be combined with the gold standard of tumor diagnosis. Thus, the diagnostic strategy based on H&E staining can be utilized to study tumors spatially. With this approach, it is possible to classify the tumor stage based on natural 3D boundary information to judge tumor invasion, which can provide an accurate clinical tumor diagnosis. Meanwhile, this approach can be used to better understand tumor metastasis and infiltration at a large scale based on mouse models.

Conventional H&E staining has been used by pathologists for more than 100 years, but trials on whole-tissue H&E staining are few^22–24^. The conventional method be utilized to obtain uniform slices rapidly, and the thickness of the tissue block is no more than 1 mm. Staining of tissues as large as a mouse brain is rarely performed because it is difficult to apply traditional H&E staining to intact tissue without any alterations. First, staining of intact tissues is usually very slow; thus, the staining speed must be accelerated. Second, for regressive staining such as H&E staining, rinsing is a key procedure. After rinsing, the morphology of the cell nucleus and cytoplasm tends to clear. However, for intact tissues, rinsing is as difficult as staining. Third, the homogeneity of staining presents other difficulties. Under normal circumstances, dyes tend to be enriched in the tissue surface, while the tissue core often shows poor dye staining. To overcome these difficulties, we adopted two strategies: the first is delipidation^25^, as tissues become porous after delipidation and are easy to stain; the second is ultrasound^26^, which can enhance the homogeneity of dye distribution in tissues. In addition, rinsing can be accelerated with this approach.

Hematoxylin can be sorted by the dye content into Harris, Ehrlich, Mayer and Gill, and among them Harris’ hematoxylin is most frequently combined with eosin to achieve counterstaining. Additionally, using this Harris’ hematoxylin, the staining time is shorter, and the cell nucleus tends to be well delineated and crisp after staining^27–29^.

In this paper, based on Harris’ hematoxylin, we propose a modified H&E staining method, termed iHE (intact tissue Hematoxylin and Eosin staining). We stained intact normal tissues and tumor tissues using iHE, and both tissue types could be stained and displayed uniform staining in the core and surface of tissues. The staining color was comparable to that of traditional H&E staining. Thus, combining the classical standard of tumor diagnosis with volume imaging can be an effective approach. Because blood vessel changes accompany tumor growth^30^, we combined blood vessel staining with iHE to provide more information on tumors in mouse models to study the metastasis and infiltration of tumors.

## Materials and Methods

### Experimental device

**Figure 1:**
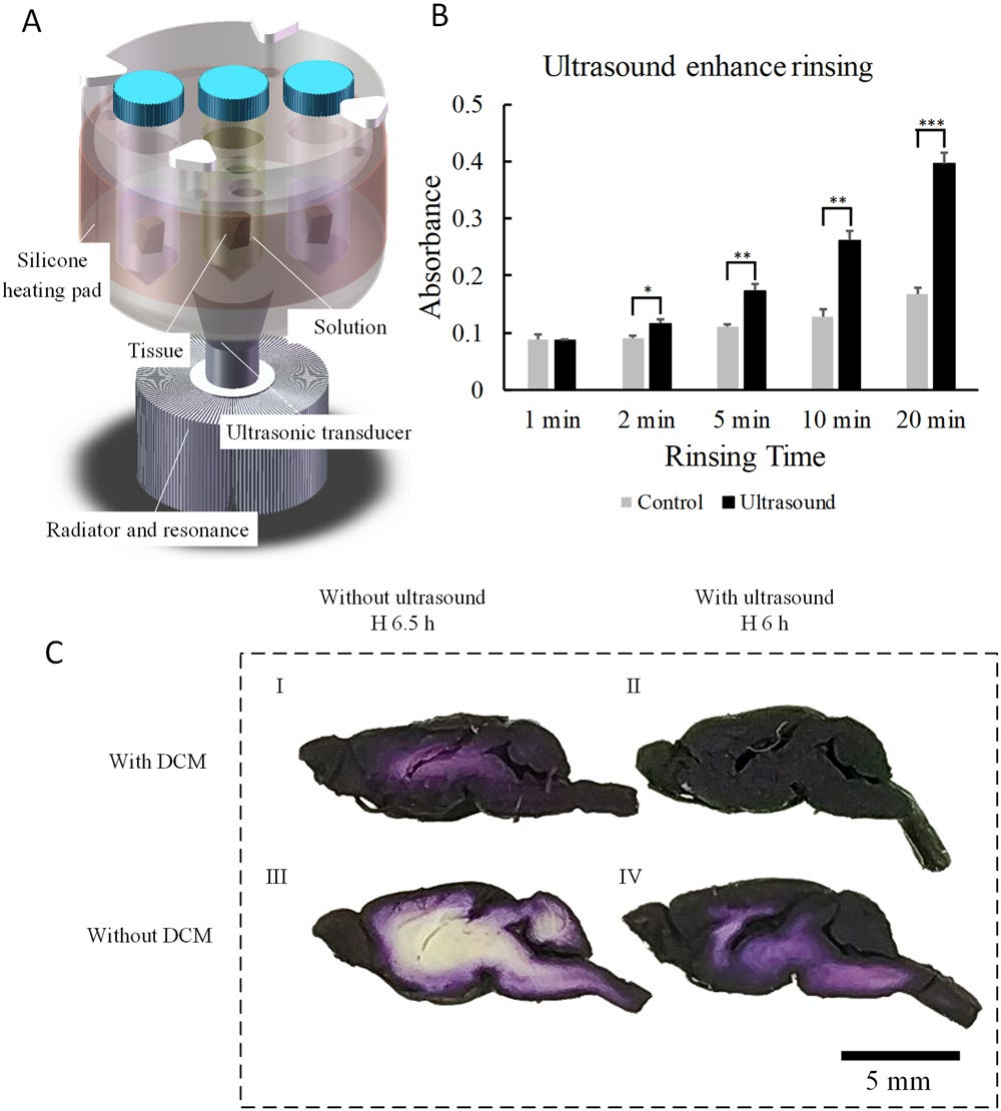
Effectiveness of iHE. (**A**) Device configuration of iHE. (**B**) Effect of ultrasound on enhanced rinsing: the x-axis represents the rinsing time, and the y-axis represents the absorbance of the rinsing solution. The p value between the ultrasound and control groups showed a sharp decrease over time (Student’s t test, n = 5). After rinsing for 20 min, the difference between the ultrasound and control groups was significant (p < 0.001). Comparison of the two groups was performed by unpaired t-test (two tailed). All data are presented as the mean ± standard error of the mean. *p < 0.05, **p < 0.01, ***p < 0.001. (**C**) Enhancing the staining effect of DCM delipidation and ultrasound. C57BL/6 mouse brains were stained with hematoxylin and then were cut along the maximum sagittal plane. I With DCM delipidation but without ultrasound; staining for 6.5 h. II With DCM delipidation and with ultrasound; staining for 6 h. III Without DCM delipidation and without ultrasound; staining for 6.5 h. IV Without DCM delipidation but with ultrasound; staining for 6 h. All staining was performed at 50 °C.

A stainless-steel container was mounted on an ultrasonic transducer, and an aluminum alloy radiator was mounted under the ultrasonic transducer to ensure rapid heat exchange (Supplementary Figs S1–3). Plastic centrifuge tubes were installed in the stainless-steel container (Fig. 1A). The container was filled with water to ensure effective propagation of ultrasound waves to tissues. The stainless-steel container was wrapped using a silicone heating pad to ensure a stable solution temperature in the plastic centrifuge tubes.

### Animals

The animal experiments were approved by the Institutional Animal Ethics Committee of Huazhong University of Science and Technology, and all experiments were performed in accordance with relevant guidelines and regulations. C57BL/6 adult mice were anesthetized with 5% chloral hydrate and 5% urethane dissolved in 0.01 M PBS solution (10 ml/kg body weight via intraperitoneal injection, 3~5 min before perfusion). Cardiac perfusion was performed with 0.01 M PBS solution and 4% paraformaldehyde solution. If blood vessel staining was needed, then perfusion was performed using carbon ink solution (20% carbon ink diluted in 0.01 M PBS solution containing 40% acrylamide and 5% bis-acrylamide); azo diisopropyl imidazoline hydrochloride served as the initiator. Next, the mice were placed at 37 °C for 2 h. If blood vessel staining was not needed, the mouse organs were separated from the mouse corpse directly after the perfusion of 4% paraformaldehyde. The mouse organs were then placed into 4% paraformaldehyde solution for 12 h.

### Delipidation

All tissues were processed under ultrasound. The mouse tissues were dehydrated with 75%, 95% and absolute ethanol each for 1.5 h at 60–70 °C. Next, the tissues were soaked in 40 °C dichloromethane (DCM) for 4 h and then rehydrated with absolute ethanol, 95% ethanol, 75% ethanol and distilled water for 1 h, 0.5 h, 1 h and 1 h at 60–70 °C.

### H&E staining

First, the tissues were stained with Harris’ hematoxylin solution for 6 h at a temperature of 60–70 °C and were then rinsed in tap water until the water was colorless. Next, 10% acetic acid and 85% ethanol in water were used to differentiate the tissue 2 times for 2 h and 10 h, and the tissues were rinsed with tap water. In the bluing step, we soaked the tissue in saturated lithium carbonate solution for 12 h and then rinsed it with tap water. Finally, staining was performed with eosin Y ethanol solution for 48 h.

### Paraffin embedding

The tissues were dehydrated with 95% ethanol twice for 0.5 h, and then soaked in xylene for 1 h at 60–70 °C followed by paraffin for 12 h. For the mouse brains, we used 0.5 mL of 95% ethanol in dehydration.

### Slicing and imaging

The stained tissues were cut into 7-μm slices, dewaxed, mounted with neutral balsam and then imaged using Nikon NIS-Elements microscopy.

## Results and Discussion

### Effectiveness of iHE

We established a simple but effective staining system (Fig. 1A). The tissues were immersed in dye solution in a centrifuge tube, which was fixed in a stainless-steel container. An ultrasonic transducer was adhered to the underside of a stainless-steel container and a radiator, which was used to cool the ultrasonic transducer. The stainless-steel container was filled with water to ensure better ultrasound transmission. The maximum electric power of the ultrasonic transducer was 60 watts. During the process of staining the tissue with hematoxylin or rinsing, we adjusted the input voltage of the ultrasonic power supply and controlled the acoustic power density in the stainless-steel container at 1.2~1.5 W/cm^2^. During tissue staining with eosin, the acoustic power density was 0.8~0.9 W/cm^2^.

Generally, it is difficult to stain intact tissue uniformly (Fig. 1C). Here, we used two means to enhance dyeing—DCM delipidation and ultrasound. DCM is a solvent that can dissolve the lipids in the cell membrane easily^5,11,15,31^. A comparison of Fig. 1C-I with Fig. 1C-III shows that the brain treated with DCM delipidation could be stained more deeply, likely because the brain becomes porous and dye diffusion tends to be enhanced after delipidation. A comparison of Fig. 1C-III with Fig. 1C-IV shows that the brain stained under ultrasound had more uniform staining in a shorter time. In Fig. 1C-II, the image shows that the brain tissue treated with DCM delipidation and stained under ultrasound for 6 h presented uniform hematoxylin staining. In other words, delipidation and ultrasound were indispensable for iHE, and both contributed to the staining results. Comparing Fig. 1C-II with Fig. 1C-III, we also found that encephalocele of the brain with ultrasound and delipidation was larger than that of the brain without ultrasound and delipidation, possibly due to the shrinkage of the brain during dehydration and delipidation.

Another effect of ultrasound was accelerated rinsing (Fig. 1B). Adult mouse brains with delipidation were first stained by hematoxylin for 6 h and then cut at the midline. One half was rinsed under ultrasound, while the other half was rinsed without ultrasound. The rinsing temperature was 50 °C. To obtain the absorbance of the ultrasound rinsing solution and static rinsing solution (control), we transferred the upper solution and measured the absorbance using the Lambda 950 UV/VIS spectrometer at a wavelength of 445 nm. Over time, the absorbance of the ultrasound rinsing solution became much higher than that of the static rinsing solution. Thus, the brain under ultrasound released more dyes than that in the static state.

### Comparison of iHE with traditional H&E staining

**Figure 2:**
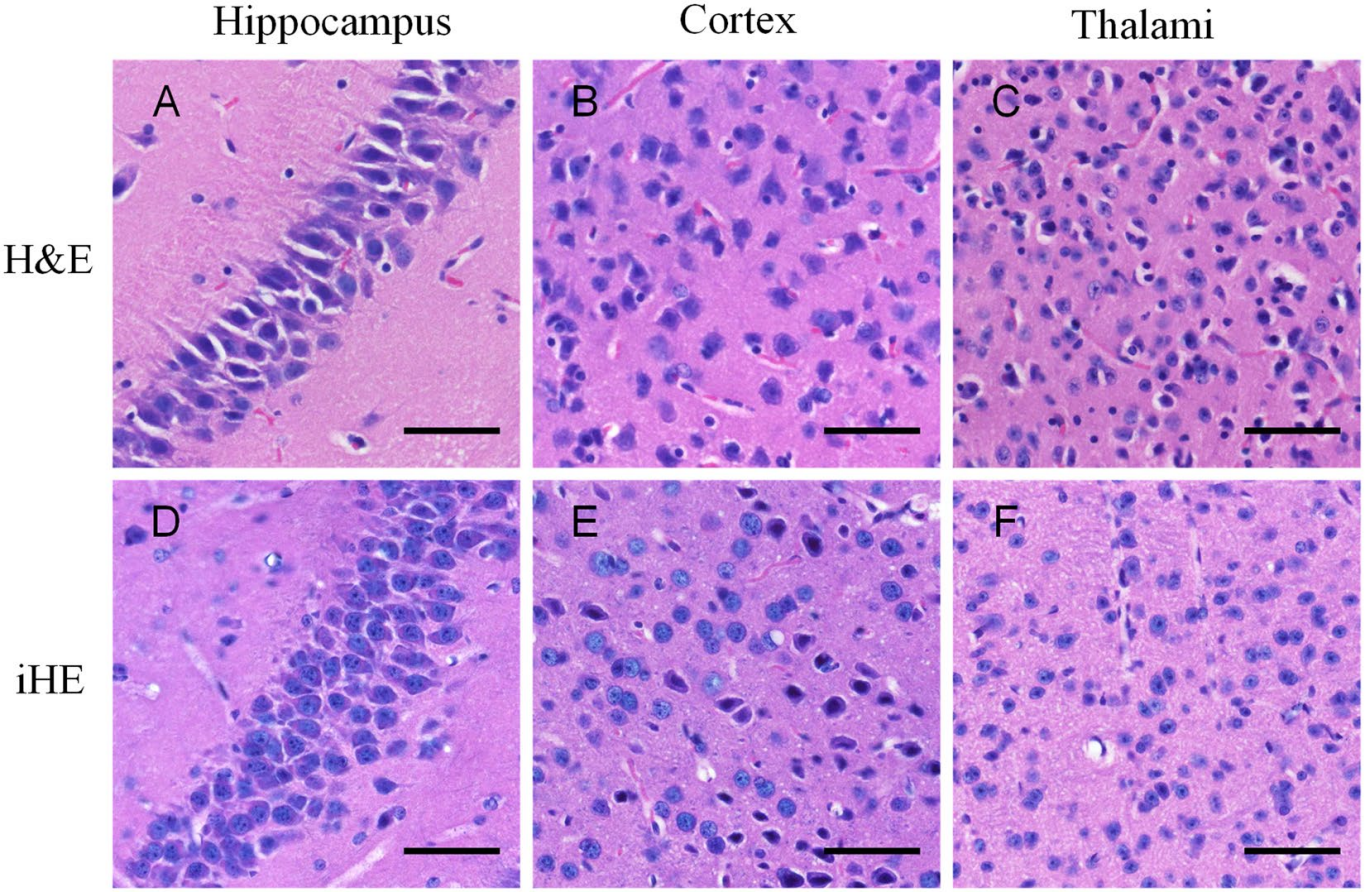
Comparison of iHE and traditional H&E staining. (**A–C**), Images of 7-μm-thick mouse brain slices stained using the traditional H&E method. (**D–F**), Images of intact mouse brain tissues after iHE staining, slicing and 2D imaging. The red matter appearing in (**A**~**F**) represents blood cells that were not completely cleared during cardiac perfusion. Objective lens, 20×; N.A., 0.75; work distance, 1 mm. Scale bar: 50 μm.

Ultrasound can lead to tissue and cell disruption; thus, a non-negligible issue is whether the cell structure is destroyed after iHE. As shown in Fig. 2, we found that the cell structure was preserved without significant distortion compared with that of traditional H&E staining. The staining effects of iHE and traditional H&E staining similar, indicating that iHE is a feasible method. The perisomatic nerve fibers shown in Fig. 2D are fewer than those in Fig. 2A, likely because the cytomembrane was partially dissolved after delipidation. To further evaluate the sacrifice of subcellular structure, we compared 100 neuron nuclei from the cortex using the H&E and iHE methods, but we found no significant difference between these two methods (data not shown).

### Intact mouse brain stained with iHE

**Figure 3:**
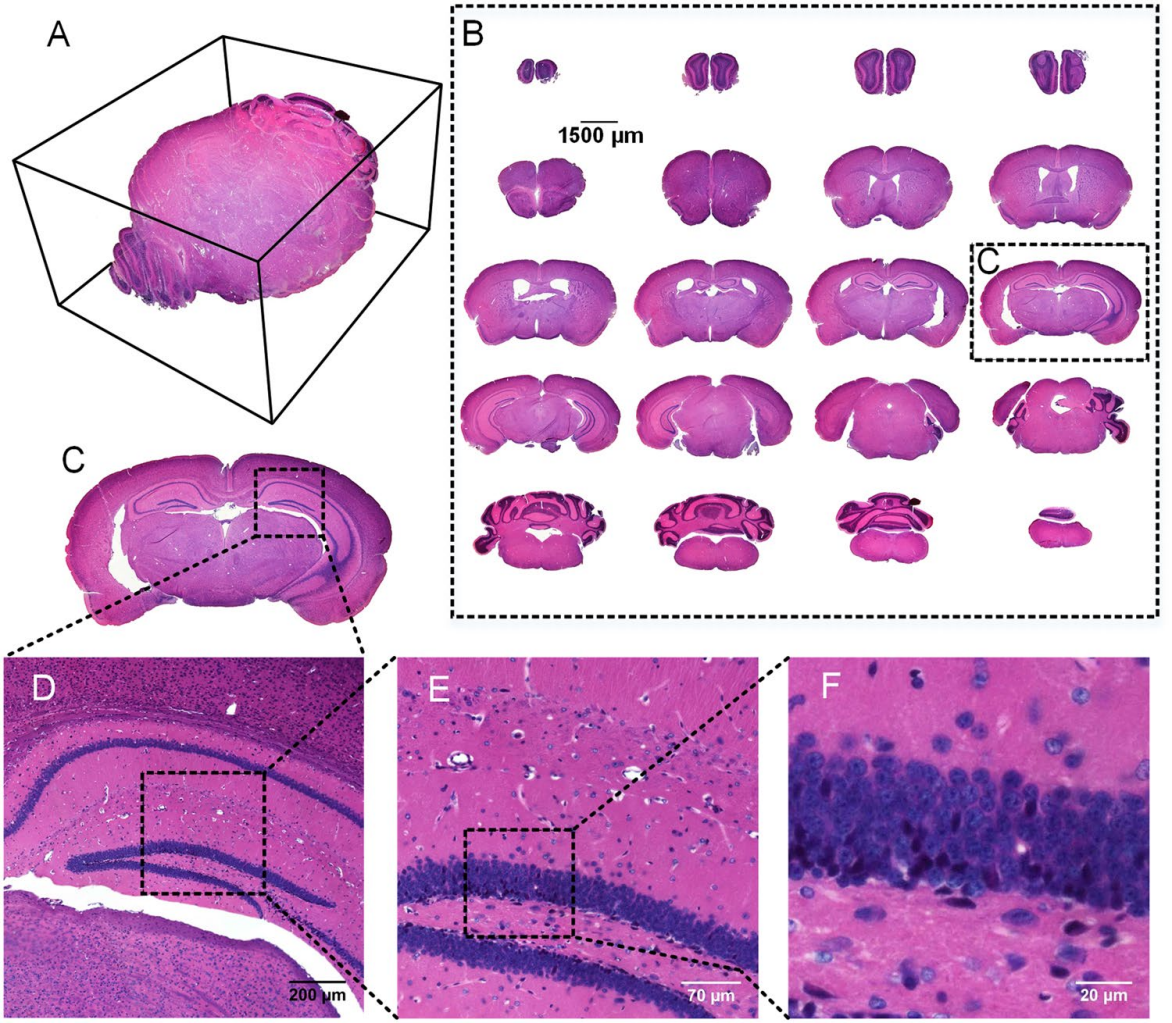
Slices from an intact mouse brain stained with iHE, followed by 2D imaging. (**A**) 3D projection of 20 slices in (**B**). (**B**) The coronal plane slices from the C57BL/6 mouse brain after iHE. Objective lens, 4× and 20×; N.A., 0.2 and 0.75. The brain was sliced at the coronal plane for 8 μm, and we selected one slice for every 400 μm from the olfactory bulb to the epencephalon. (**D**–**F**) Magnification of (**C**).

An intact C57BL/6 mouse brain was stained with iHE, and the images in Fig. 3 show that the brain was stained uniformly. The hippocampus was distinguishable, and the cellular nuclei morphology was clear (Fig. 3D–F). Thus, we could acquire H&E staining information for a mouse brain based on its natural spatial context. Encephalocoele of the brain was apparent (Fig. 3C).

### Other intact mouse tissues stained with iHE

**Figure 4:**
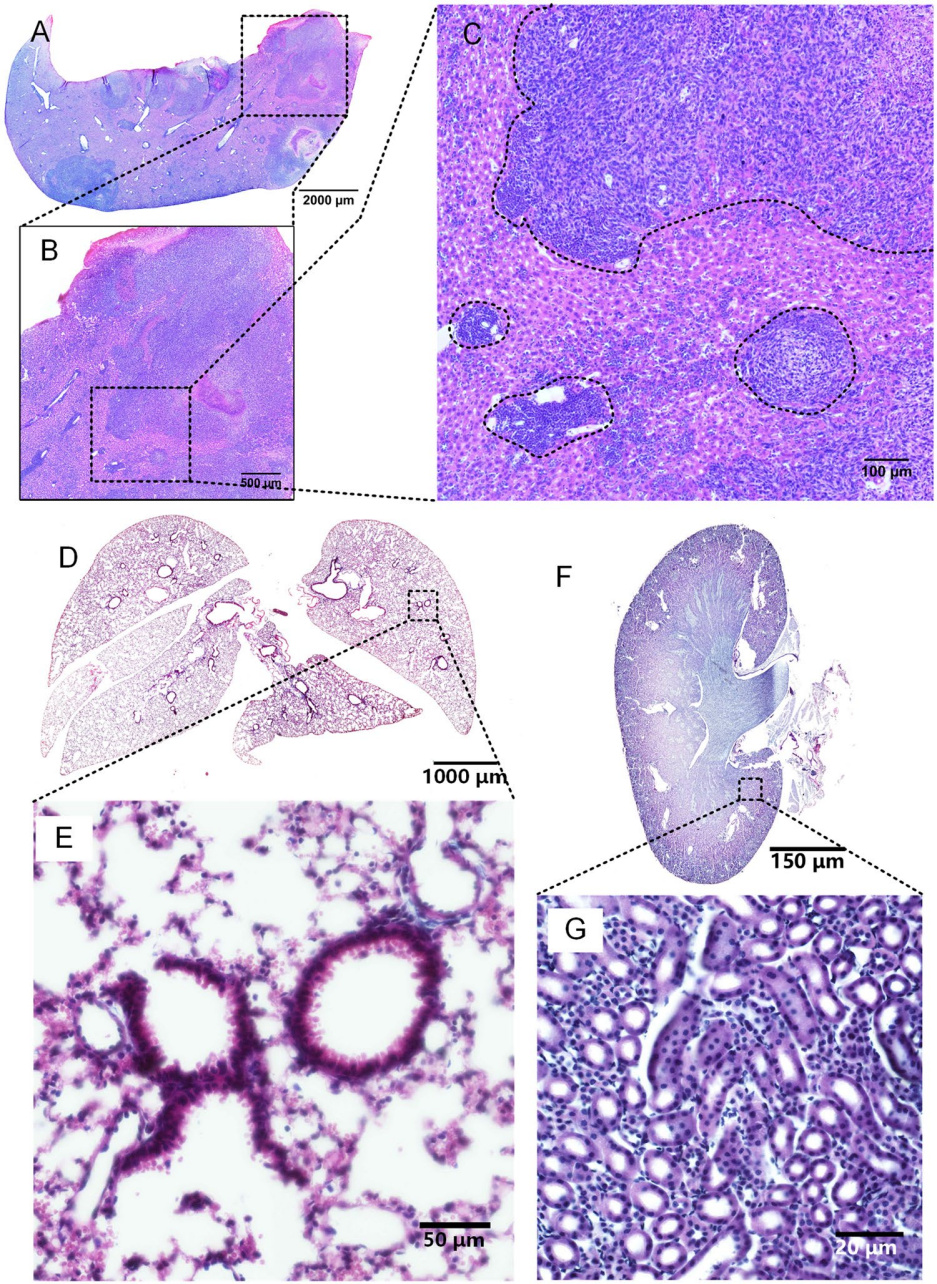
Images of other mouse tissues stained with iHE. (**A**–**C**) Images of mouse liver with a tumor. (**D**,**E**) iHE for mouse lung. (**F**,**G**) iHE for mouse kidney. Objective lens, 20×; N.A., 0.75; work distance, 1 mm.

To generate a liver metastasis model, we injected 4T-1 mammary cancer cells into 7-week-old BALB/c mice, reared the mice for 40 days and perfused the mice at 3 months of age. Next, the liver was stained, and slices were prepared for subsequent imaging using Nikon NIS-Elements microscopy; the results are shown in Fig. 4A–C. In the image, the black dotted line is used to divide the tumor area and normal area, and the results show that the nuclear-cytoplasmic ratio of the tumor area was different from that of the normal area. The mouse lung, kidney, stomach, forepaw, heart and eyeballs were stained using iHE (Figs 4D–G and S4A–H). The pulmonary lobe and alveoli, classic lung structures, can be observed in Fig. 4D,E. Kidney tubules are presented clearly in Fig. 4F,G. The mouse stomach was stained, and the villus at the pylorus is shown in Supplementary Fig. 4G,H. The classical muscle structure in the mouse forepaw was revealed after iHE, and the skin microstructure was also observed (Supplementary Fig. S4A,B). The heart is an organ comprised of cardiomyocytes, and chamber and myocardial cell types were clearly observed (Supplementary Fig. S4C,D). We also applied iHE to mouse eyeballs, and the cellular stratification of the retina could be observed (Supplementary Fig. S4E,F).

### Compatibility of iHE with blood vessel staining

**Figure 5:**
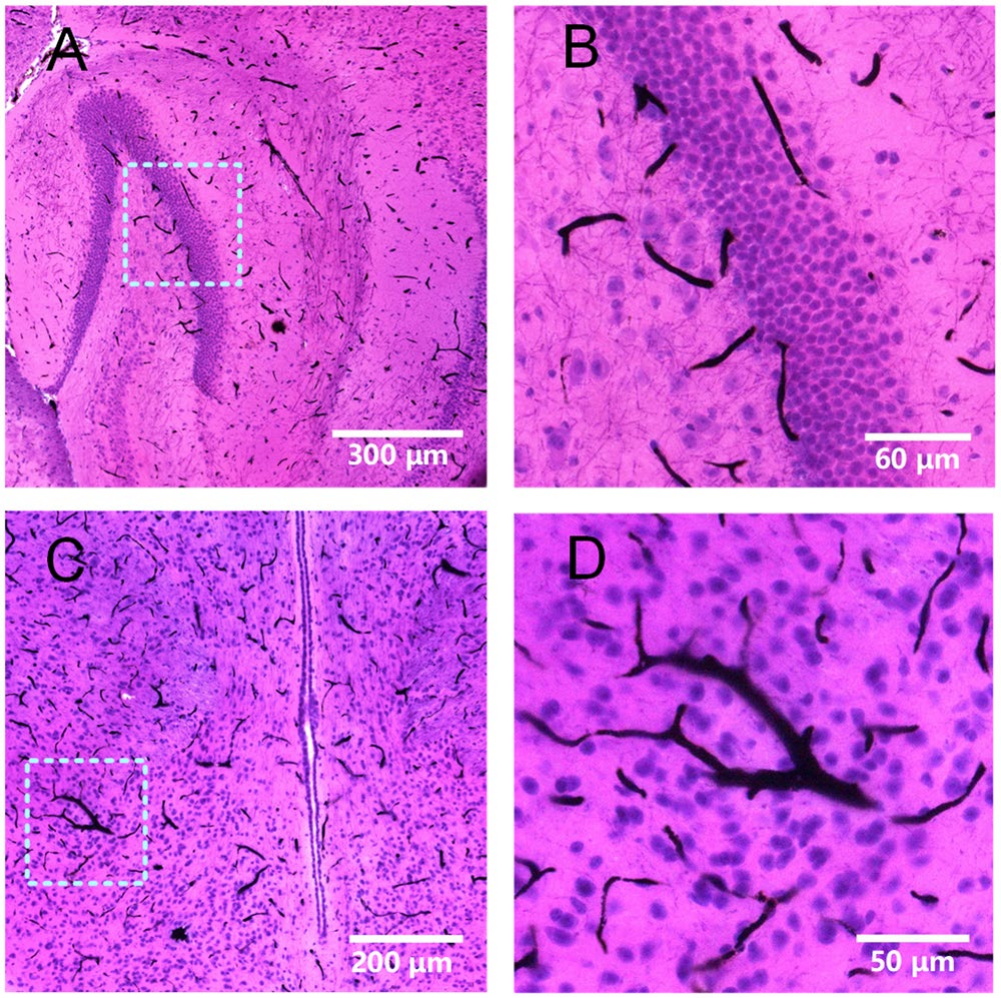
Mouse brain perfused with carbon ink before iHE. (**A**,**B**) Hippocampus, (**B**) is the magnification of the box in (**A**). (**C**,**D**) Thalami, (**D**) is the magnification of the box in (**C**). Objective lens, 20×; N.A., 0.75; work distance, 1 mm.

Cancer cell growth is associated with significant changes in blood vessels^30^. As discussed above, H&E staining is a classic means to present the details of a tumor. Here, we combined H&E staining with blood vessel staining (Fig. 5) to observe blood vessel and cellular nuclei information simultaneously. This double staining method has the potential to provide meaningful information on tumor status.

**Figure 6:**
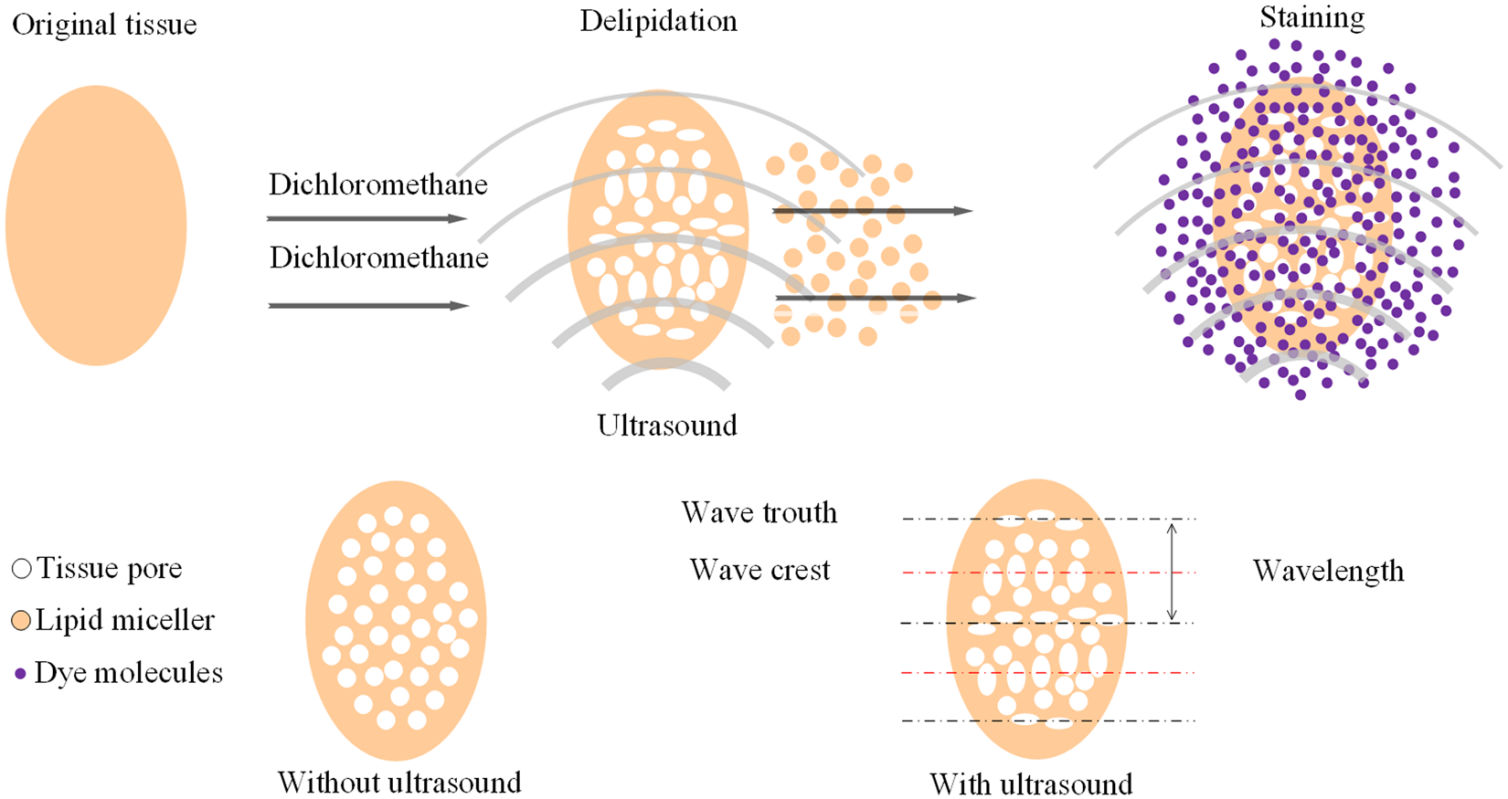
Possible principle of iHE. A fixed tissue is used as the original tissue. After dehydration, the tissue is subjected to delipidation, followed by the H&E staining procedure; the H&E staining process was simplified so that the reader could better understand the principle for how ultrasound is involved in the process. Dichloromethane can cause a porous state of the cell membrane, equal to the porous state of the tissue. The pores of the tissue change in the ultrasonic field because ultrasound is a longitudinal wave. Thus, the pores in the wave crest of the ultrasound are stretched, while the pores in the trough of the waves are compressed. Meanwhile, the random motion of particles is enhanced in the ultrasonic field. The diffusion process is enhanced and achieves rapid and uniform staining.

The principle of iHE is shown in Fig. 6. After DCM delipidation, the cytomembrane is partly dissolved, and the tissue becomes porous^25^. We assume the ultrasound causes stable cavitation (ultrasound energy density <10 Watt/cm^2^). The pores of the tissue may change their diameter in the ultrasonic field because ultrasound is a longitudinal wave. Similar to a spring stretching and compressing due to an external force, the tissue is stretched and compressed at the microscale level. Thus, the pores in the wave crest of the ultrasound are stretched, while the pores in the trough of the waves are compressed^26,32,33^. Meanwhile, with the ultrasonic wave, there is periodic compression and rarefaction in the liquid. Thus, mass transfer in and out of the tissue is facilitated^34^. Therefore, the diffusion driven by the concentration difference in the particles is accelerated in the ultrasonic field, and the dyes diffuse into tissue faster.

Overall, the iHE method creates porous tissue for staining and enhances random motion. Therefore, the diffusion process is enhanced and achieves rapid and uniform H&E staining. We established a set of methods (iHE) to obtain H&E staining information of intact tissue for volume imaging. Ultrasound was used to facilitate the staining of whole-mount tissue, and its ability to enhance rinsing was evaluated quantitatively. As a result, this method can classify the invasion stage of the tumor based on its natural 3D boundary and better elucidate tumor metastasis on a large scale based on a mouse model. iHE was also compatible with blood vessel staining, implying that we might obtain H&E staining and blood vessel information for a single tissue simultaneously. Thus, our results provide a novel tool to study tumor metastasis and infiltration in a mouse model. Furthermore, ultrasound shows promise in facilitating other types of biological tissue processing, such as optical clearing, immunolabeling and chemical staining of intact tissues.

## Electronic supplementary material

Supplementary Information
