# Supplementary material for: Hematoxylin and eosin staining of intact tissues via delipidation and ultrasound
Source: Sci Rep. 2018 Aug 16;8:12259. doi: 10.1038/s41598-018-30755-5 (PMC6095898; doi:10.1038/s41598-018-30755-5)
Supplement: Supplementary file 1 — Supplementary Information [file 41598_2018_30755_MOESM1_ESM.docx]

**Supplementary Information**

**Hematoxylin and eosin staining of intact tissues via delipidation and ultrasound**

Yawu Li^1,2†^, Ning Li^1,2†^, Kai Huang^3^, Ting Zheng^4^, Xiang Yu^1,2^, Xiaofeng Cheng^1,2^, Shaoqun Zeng^1,2^, Xiuli Liu^1,2*^

**Materials and methods**

**Staining procedure** [^1^](#_ENREF_1)^,^[^2^](#_ENREF_2)

Draw materials

C57BL/6 adult mice were anesthetized with 5% chloral hydrate and 5% urethane dissolved in 0.01 M PBS solution (10 ml/kg body weight via intraperitoneal injection, 3~5 min before perfusion). Cardiac perfusion was performed with 0.01 M PBS solution and 4% paraformaldehyde solution. If blood vessel staining was needed, then perfusion was performed using carbon ink solution (20% carbon ink diluted in 0.01 M PBS solution containing 40% acrylamide and 5% bis-acrylamide); azo diisopropyl imidazoline hydrochloride served as the initiator. Next, the mice were placed at 37°C for 2 h. If blood vessel staining was not needed, the mouse organs were separated from the mouse corpse directly after the perfusion of 4% paraformaldehyde. The mouse organs were then placed into 4% paraformaldehyde solution for 12 h.

Dehydration

All tissues were processed under ultrasound. The mouse tissues were dehydrated with 75%, 95% and absolute ethanol each for 1.5 h at 60-70°C.

Delipidation

Next the tissues were soaked in 40°C dichloromethane (DCM) for 4 h.

Rehydration

Rehydrated with absolute ethanol, 95% ethanol, 75% ethanol and distilled water for 1 h, 0.5 h, 1 h and 1 h at 60-70°C.

Haematoxylin staining

The tissues were stained with Harris' hematoxylin solution for 6 h at a temperature of 60-70°C and were then rinsed in tap water until the water was colorless.

Differentiation

10% acetic acid and 85% ethanol in water were used to differentiate the tissue 2 times for 2 h and 10 h, and the tissues were rinsed with tap water.

Bluing

In the bluing step, the tissues were soaked in saturated lithium carbonate solution for 12 h and then rinsed with tap water

Eosin staining

Staining was performed with eosin Y ethanol solution for 48 h.

Paraffin embedding

The tissues were dehydrated with 95% ethanol twice for 0.5 h, and then soaked in xylene for 1 h at 60-70°C followed by paraffin for 12 h. For the mouse brains, we used 0.5 mL of 95% ethanol.

Slicing and imaging

The stained tissues were cut into 7-μm slices, dewaxed, mounted with neutral balsam and then imaged using Nikon NIS-Elements microscopy.

**Establishment of the iHE-device**

In Figure S1, glass fiber net was used for insulation between the ultrasonic transducer and stainless-steel container. Ultrasonic transducer glue was chosen to bond the ultrasonic transducer and stainless-steel container. Aluminum alloy heat sink was mounted under the ultrasonic transducer to conduct heat from transducer to air. The power of ultrasonic transducer is 60 watts, and its frequency is 40 kHz.

Mixed glue on bottom surface of stainless-steel container (Figure S2A). Sticked double layers of glass fiber net on mixed glue, and put the ultrasonic transducer on glass fiber net (shown in Figure S2B), pressed the ultrasonic transducer with a heavy objective, like iron ingots to achieve better adhesion. Next spliced the aluminum alloy heat sink on the ultrasonic transducer. We designed a shelf to fix centrifuge tube as shown in Figure S2D.

The silicone pad (5 cm*45 cm, custom-made, orange in Figure S3 B) surrounding stainless steel container was used to heat. Put the iHE container on silicone pad to prevent it moving when working. Ultrasound power supply was home-made. SCR voltage regulator was used to control the input voltage of ultrasound power supply and the ultrasound power density in iHE container.


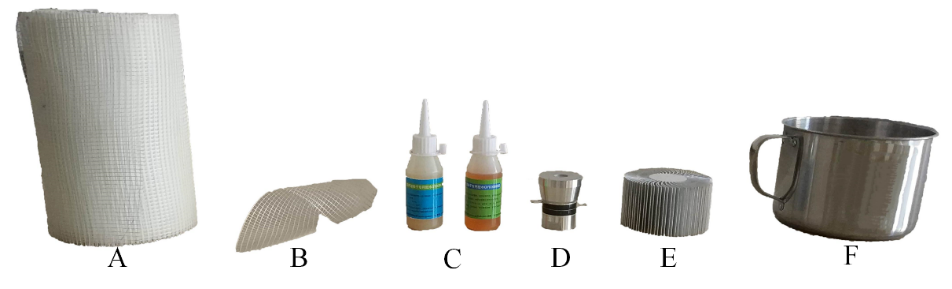


**Figure S1** | Raw materials to establish the iHE device. **(A, B)** Glass fiber net. **(C)** Ultrasonic transducer glue. **(D)** Ultrasonic transducer. **(E)** Aluminum alloy heat sink. **(F)** Stainless-steel container.


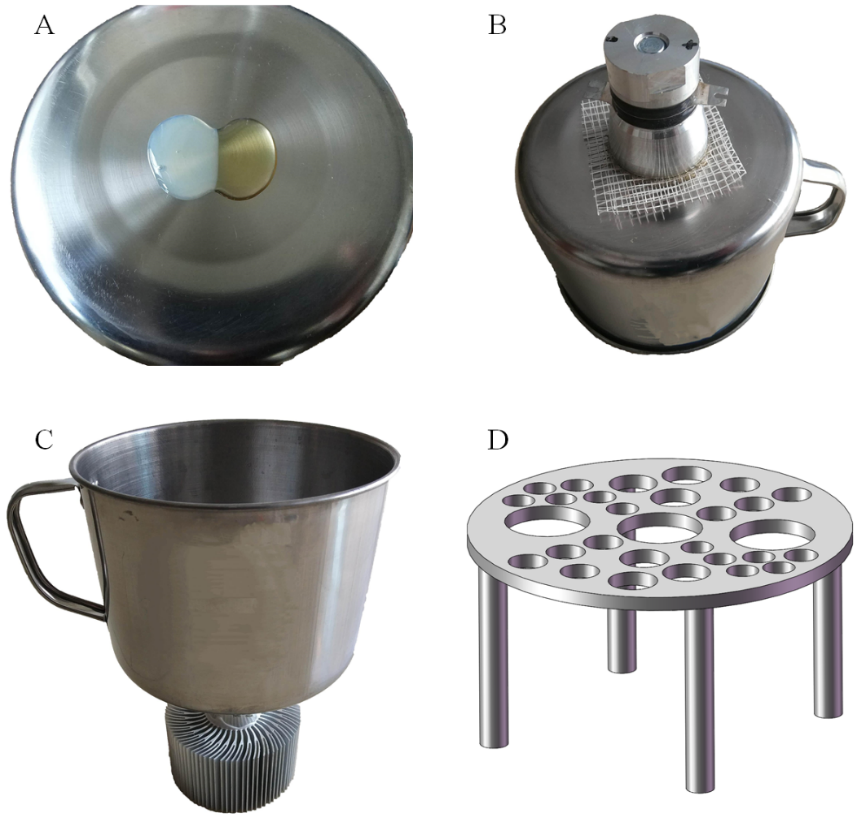


**Figure S2** | Process to build the iHE container.


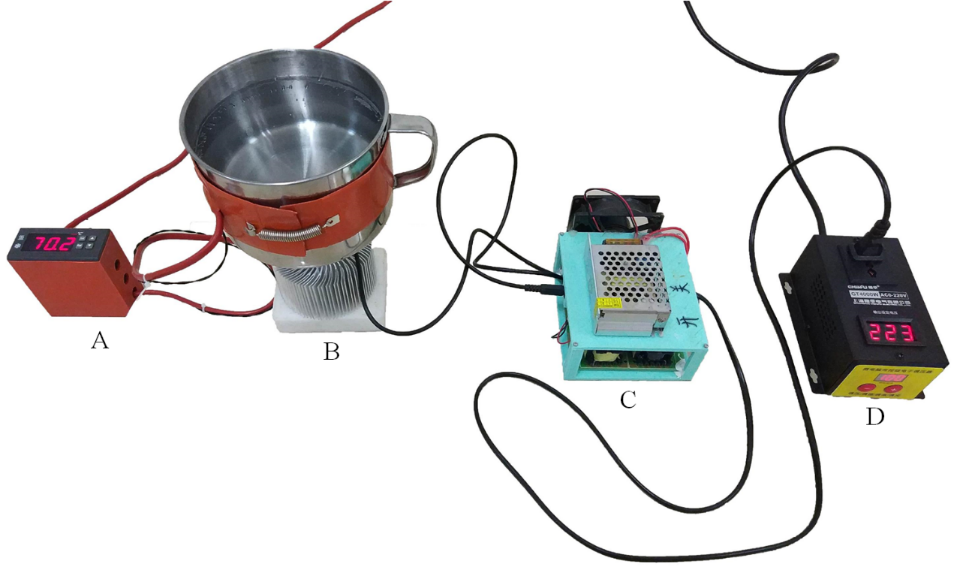


**Figure S3** | The equipment construction of the iHE system. **(A)** Temperature controller. **(B)** Silicone pad. **(C)** Ultrasound power supply. **(D)** SCR voltage regulator.

**Other application of iHE**

Other intact mouse tissues stained with iHE.

**
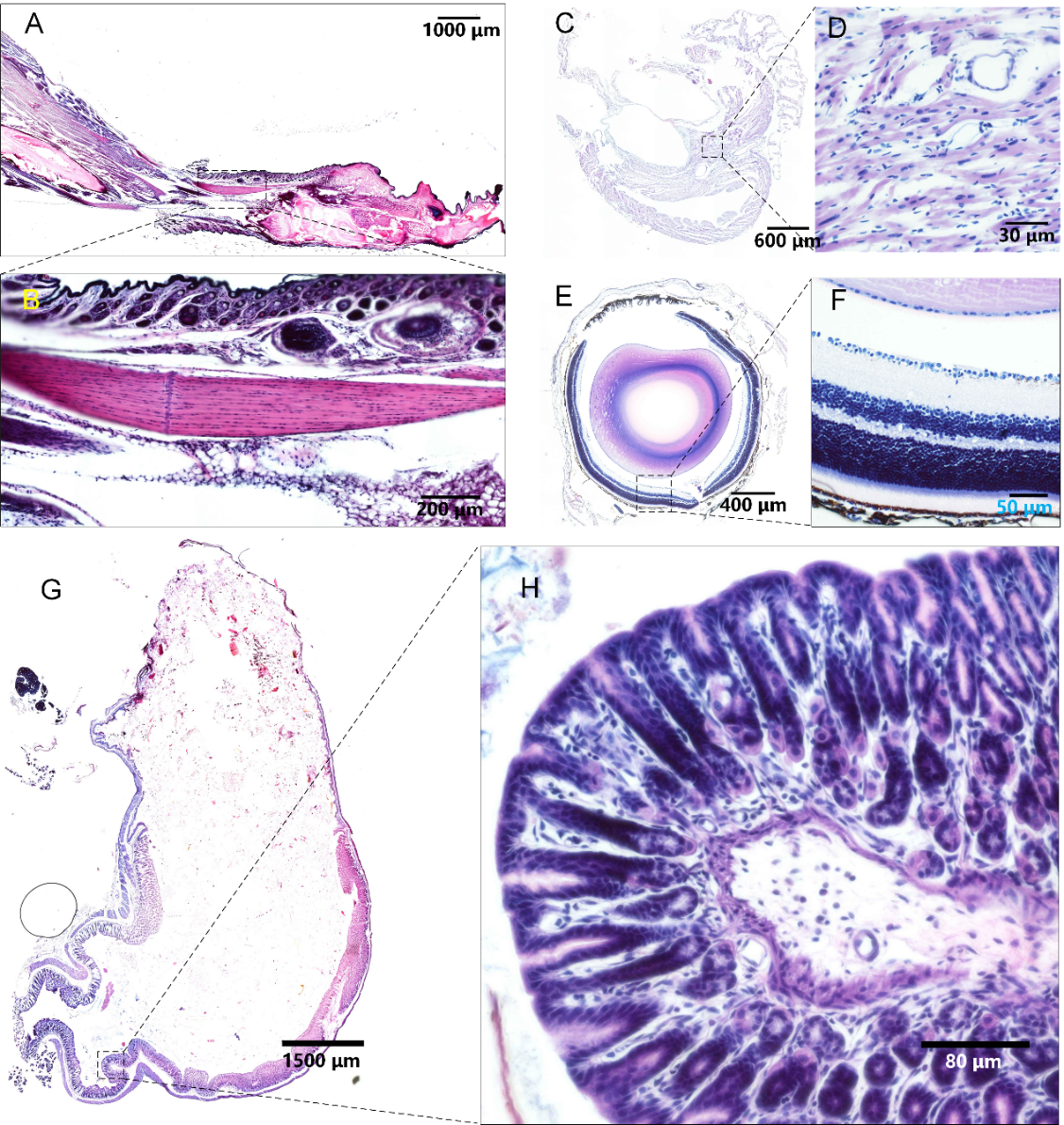
**

**Figure S4** | iHE for other mouse tissues. **(A, B)** Mouse forepaw stained with iHE. **(C, D)** Mouse heart stained with iHE. **(E, F)** Mouse eyeballs stained with iHE. All of these tissues are from adult C57BL/6 mouse. 20 × objective lens with N.A. 0.75 and working distance of 1 mm is used.

**Reference**

1. Mayer, F. & Cook, A. H. *The chemistry of natural coloring matters; the constitutions, properties, and biological relations of the important natural pigments*. (Reinhold publishing corporation, 1943).

2. Nietzki, R. *Chemie der organischen farbstoffe*. 4. verm. aufl. edn, (J. Springer, 1901).
